# Functional autoantibodies in patients with different forms of dementia

**DOI:** 10.1371/journal.pone.0192778

**Published:** 2018-03-14

**Authors:** Gerd Wallukat, Harald Prüss, Johannes Müller, Ingolf Schimke

**Affiliations:** 1 Berlin Cures GmbH, Berlin, Germany; 2 Klinik für Neurologie, Charité - Universitätsmedizin Berlin, Berlin, Germany; 3 Deutsches Zentrum für Neurodegenerative Erkrankungen (DZNE), Berlin, Germany; University of North Dakota, UNITED STATES

## Abstract

Dementia in general and Alzheimer’s disease in particular is increasingly seen in association with autoimmunity being causatively or supportively involved in the pathogenesis. Besides classic autoantibodies (AABs) present in dementia patients, there is the new autoantibody class called functional autoantibodies, which is directed against G-protein coupled receptors (GPCRs; GPCR-AABs) and are seen as pathogenic players. However, less is known about dementia patients’ burden with functional autoantibodies. We present here for the first time a study analyzing the prevalence of GPCR-AABs in patients with different dementia forms such as unclassified, Lewy body, vascular and Alzheimer’s dementia. We identified the GPCR-AABs’ specific targets on the receptors and introduced a neutralization strategy for GPCR-AABs. Patients with Alzheimer’s and vascular dementia carried GPCR-AABs targeting the first loop of the alpha1- and the second loop of the beta2-adrenergic receptors (α1-AABs; β2-AABs). Nearly all vascular dementia patients also carry autoantibodies targeting the endothelin A receptor (ETA-AABs). The majority of patients with Lewy body dementia lacked any of the GPCR-AABs. *In vitro*, the function of the dementia-associated GPCR-AABs could be neutralized by the aptamer BC007. Due to the presence of GPCR-AABs in dementia patients mainly in those suffering from Alzheimer’s and vascular dementia, the orchestra of immune players in these dementia forms, so far preferentially represented by the classic autoantibodies, should be supplemented by functional autoantibodies. As dementia-associated functional autoantibodies could be neutralized by the aptamer BC007, the first step was taken for a new *in vivo* treatment option in dementia patients who were positive for GPCR-AABs.

## Introduction

Dementia in general and particularly Alzheimer’s disease are seen increasingly in association with an autoimmune background that could be causatively or supportively involved in the pathogenesis. In addition to the variety of autoantibodies (AABs) detected in patients with dementia and suggested to be pathogenic players, biomarkers and treatment targets such as those summarized in [1,2], there is a new class of autoantibodies, the so-called functional autoantibodies that are directed against G-protein coupled receptors (GPCRs; GPCR-AABs) which are increasingly seen as pathogenic players. For GPCR-AABs and the related diseases, which can be named “functional autoantibody disease”, basics, diagnostics and treatment strategies are summarized in [3,4,5,6]. In patients with dementia, GPCR-AABs targeting α1- and β2-adrenergic receptors (α1-AABs; β2-AABs) [7,8], as well as the angiotensin 2 type 1 receptor (AT1-AABs) [9], have already been demonstrated, which possibly links dementia to the specific autoimmune background of functional autoantibody disease. However, data related to the different dementia forms are missing for these GPCR-AABs and for further vasoactive GPCR-AABs, specifically those directed against the endothelin A receptor (ETA-R, ETA-AABs), which could additionally affect dementia patients. Here, we present for the first time a study analyzing the GPCR-AAB prevalence in patients with different forms of dementia. We found significantly higher frequencies for α1-, β2- and ETA-AABs in patient with vascular dementia compared to patients with Alzheimer’s disease and even more with unclassified dementia, where ETA-AABs were widely missed. Patients with Lewy body dementia lacked GPCR-AABs in a very high percentage. AT1-AABs were absent in all patient groups. Additionally, we present the GPCR-AABs’ target regions on the receptors as well as the possibility to neutralize dementia-associated GPCR-AABs by the aptamer BC007 [10].

## Material and methods

### Patients

Sera were primarily sampled for the study “High prevalence of NMDA receptor IgA/IgM antibodies in different dementia types” [11]. For this retrospective descriptive subgroup analysis to analyze the prevalence of GPCR-AABs in patients with different forms of dementia, serum were used (based on the availability in quantities necessary for the GPCR-AAB analysis) of patients with unclassified, Lewy body, vascular, and Alzheimer’s dementia attending the Department of Neurology, Charité—Universitätsmedizin Berlin. For patients’ basic data, group composition, comorbidities, and medication, see Table 1 in results. The study was approved by the institutional Review Board of Charité–Universitätsmedizin Berlin; written informed consent was obtained from patients or their representatives.

### GPCR-AAB analytics

To identify and quantify the GPCR-AABs, a bioassay established by Wallukat and Wollenberger was used [12], which was modified and standardized as described in [13,14]. In this bioassay, the chronotropic response of spontaneously beating cultured neonatal rat cardiomyocytes to patients’ IgG-containing GPCR-AABs was recorded.

#### Bioassay of spontaneously beating cultured neonatal rat cardiomyocytes

**Fig 1:**
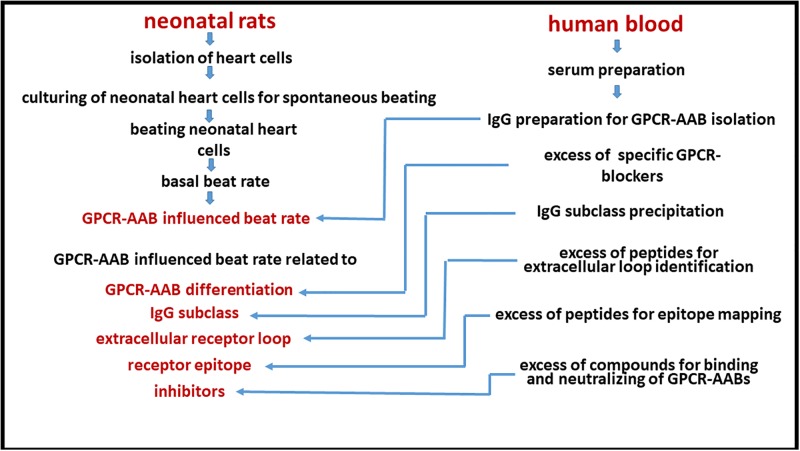
Chart of the bioassay using spontaneously beating cultured neonatal rat cardiomyocytes for the characterization of autoantibodies directed against G-protein-coupled receptors (GPCR-AABs).

As schematically illustrated in Fig 1, to investigate GPCR-AABs, IgG was isolated from patient serum, which is the sample material required for the bioassay of spontaneously beating cultured neonatal rat cardiomyocytes. This bioassay measured the functional activity of the GPCRs via the cells’ chronotropic response after addition of the GPCR-AAB-containing IgG. Depending upon either the positive or negative chronotropic activity of the GPCR-AABs, the increase and decrease, respectively, of the cells’ beat frequency is monitored.

With the intelligent use of blockers and competitors, the GPCR-AABs are specified for their targeted receptors, extracellular binding sites and specific receptor epitopes, as well as to analyze any functionality activity change of the GPCR-AABs.

#### Sample preparation

After whole blood collection, serum was prepared according to standardized procedures. For the IgG preparation, 1 ml of serum and 660 μl of saturated ammonium sulfate solution was mixed (final concentration: 40% ammonium sulfate) and incubated for 18 h at 4°C. After centrifugation for 15 min at 6,000 *g*, the pellet was re-suspended in 750 μl of PBS, mixed with 750 μl of saturated ammonium sulfate solution (final concentration: 50% ammonium sulfate), and centrifuged again. Thereafter, the pellet was suspended in 700 μl of PBS and dialyzed (VISKING cellulose, type 27/32, MW Cut off 14 kDa; Carl Roth, Germany) against the 100-fold volume of PBS for 3 days at 4°C. The resulting IgG fraction was aliquotted and stored at −20°C for at least a month without a loss of activity.

#### Cardiomyocyte preparation and culturing

Hearts of approximately twenty 1- to 3-day-old rats were removed under sterile conditions and transferred to PBS (4°C; without Ca2+, Mg 2+; Biochrom, Berlin, Germany). The ventricle tissue was separated and dissected into small pieces of nearly of nearly 1 mm^3^ for washing twice with 10 ml of solution 1. After decanting the wash solution, the tissue was re-suspended in 10 ml of PBS containing 0.2% of crude trypsin, and incubated for 15 min at 37°C under stirring; thereafter, the solution was treated with 10 ml of ice-cold heat-inactivated calf serum to stop the trypsination. The resulting suspension was centrifuged at 130 *g* for 6 min and the pellet was transferred to 20 ml of SM20-I medium (Biochrom GmbH, Berlin, Germany). For cell counting, 100 μl of this suspension was added to 100 Trypan blue solution. Then, 2.4×10^6^ cells in 2.0 mL of SM 20-I medium, which was equilibrated with humid air, were transferred to 12.5cm^2^ Falcon flasks, and cultured as a monolayer for 4–8 days at 37°C. The medium was renewed after 2 days. Cardiomyocytes spontaneously started beating after 2 days in culture.

#### Assay procedure and standardization

On the day of measurement, the culture flask was transferred onto a heated stage (37°C) microscope and 6 fields with synchronic and rhythmic beating cardiomyocytes were marked on the flask bottom. Thereafter, the basal beating rate of the 6 fields was counted for 15 seconds and averaged. After the addition of 40 μl of the IgG preparation, the culture flasks were incubated for 40 to 60 min at 37°C and the beating rate in the 6 fields was then counted for 15 sec and averaged.

For GPCR-AAB measurements, the bioassay has to fulfill the following quality criteria: 1) the basal beating rate must range between 120 and 160 beats/min; 2) cells stimulated for 5 min with isoprenaline (10 μM) must respond with a frequency increase of more than 45 beats/min; and 3) cells must respond to goat anti-ADRB1 (0.5 μg/ml, 1:100), EB07133, a commercial polyclonal antibody against the beta1-adrenergic receptor (Everest Biotech Ltd., Oxfordshire, United Kingdom), with an increase in frequency of more than 20 beats/min after incubation for 1 h. For the delta beating rate in the presence of isoprenaline and the commercial autoantibody, a day-to-day variation of ≤15% was estimated.

#### Calculation of the GPCR-AAB activity

One unit of GPCR-AAB activity corresponds to a 1 beat/min frequency change. The lower limits of detection (LLD) for positive and negative chronotropic activity were calculated as 4.0 U and -4.0 U, respectively. GPCR-AAB positivity was defined using cut-offs based on x ± 3 SD of the GPCR-AAB level of more than 100 healthy subjects. Results of ≥ 8.0 U for positive and ≤ -8.0 U for negative chronotropic GPCR-AAB activity were calculated. To access any integrated autoimmune activity for the several patient groups, we calculated a score based on the general GPCR-AAB composition in the groups for which the GPCR-AAB activities were summarized based on: 0 point = GPCR-AAB level <lower limit of detection (LLD); 1 point = GPCR-AAB level > LLD < cut off; and 2 points = GPCR-AAB level > cut off.

#### GPCR-AAB differentiation related to their targeted receptors

**Fig 2:**
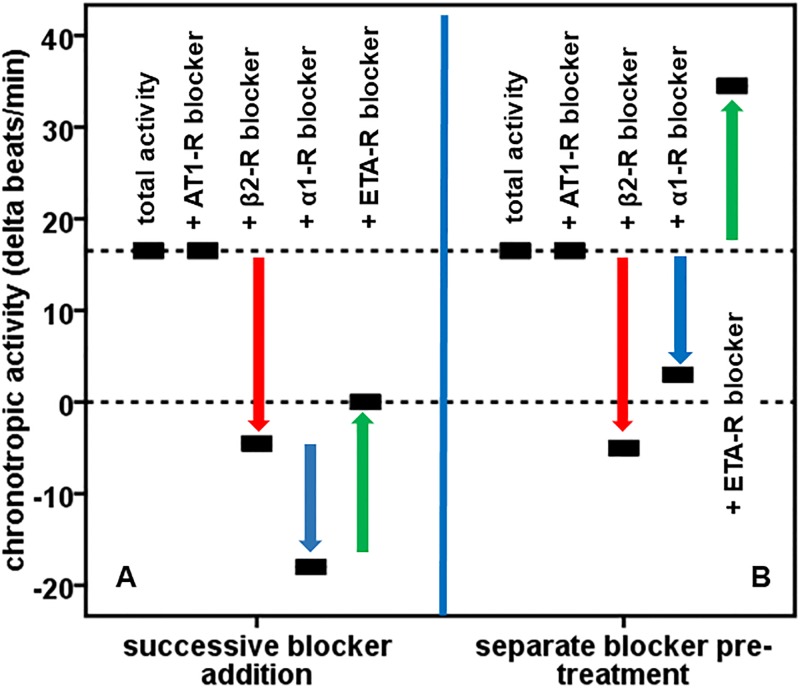
Measurement strategy for autoantibodies directed against G-protein coupled receptors (GPCR-AABs) in patients’ IgG using the bioassay of cultured spontaneously beating neonatal rat cardiomyocytes. The IgGs’ chronotropic activity resulting from the presence of positive and negative chronotropic GPCR-AABs was monitored. For GPCR-AAB differentiation and activity calculation, the bioassay was performed after the successive addition of blockers against the AT1 (0.1 μmol/l Losartan), β2-adrenergic (0.1 μmol/l ICI 118551), α1-adrenergic (0.1 μmol/l Prazosin) and endothelin A receptors (0.1 μmol/l BQ123) and (for independent confirmation) after IgG pre-treatment separately with the respective blockers.

Through the use of specific blockers of GPCRs, the cells’ chronotropic response can be attributed to the related GPCR-AABs. Using this strategy, the bioassay was performed in the presence of specific blockers for the α1-adrenergic (0.1 μmol/l Prazosin), β2-adrenergic (0.1 μm/l ICI 118.551), ETA receptor (0.1 μmol/l BQ 123) and AT1 receptors (0.1 μmol/l Losartan). Fig 2 illustrates this measurement strategy representatively for one patient positive for α1-, β2-, and ETA-AABs and negative for AT1-AABs. Due to the successive addition of receptor blockers, the change in the cells’ beating rate, which is the result of positive (α1-AABs, β2-AABs, AT1-AABs) and negative chronotropy (ETA-AABs), can be attributed to the individual GPCR-AABs (A). For independent confirmation, the IgGs were pre-treated separately with each blocker before the GPCR-AAB measurement (B).

#### Localization of the extracellular receptor binding side of the GPCR-AABs

To localize the extracellular binding side (loops), 50 μl of the GPCR-AAB-containing IgG preparation was pre-incubated for 30 min with 2 μl of solutions containing synthetic peptides (50 μmol/l) (Biosyntan GmbH, Berlin-Buch, Germany) which represent the first, second and third extracellular loops of β2-adrenergic, α1-adrenergic and ETA receptors. Then, 40 ml of this mixture was added to the Bioassay for GPCR-AAB measurements. To localize the extracellular binding side of the ETA-AABs described here first, (^134^LPINVFKLLAGRWPFDHNDFGVFLCKL^160^), (^229^FEYRGEQHKTCMLNATSKFMEFYQDVKD^256^), and (^329^KKTVYNEMDKNRCELLLSFLL^348^), respectively, were used which represent the first, second and third extracellular loop ETA receptors.

#### Mapping of the specific epitopes targeted by the GPCR-AABs

To map the specific epitope on the receptor loop targeted by the GPCR-AABs, the bioassay was performed after pre-treatment of the GPCR-AABs with an excess of synthetic peptides (Biosyntan GmbH, Berlin-Buch, Germany), which overlapped to represent the amino acid sequence of the receptor loops, first described for GPCR-AABs against the beta1-adrenergic receptor in [15]. For the mapping of the ETA-AAB epitope on the second extracellular loop demonstrated here, peptides were used, as follows: P1: FEYRGEQ, P2: EQHKTCM, P3: MLNATSK, P4: SKFMEFY, and P5: FYQDVKD. For this, 50 μl of the GPCR-AAB-containing IgG preparation was pre-incubated for 30 min with 2 μl of solutions containing the synthetic peptides (100 μmol/l) (Biosyntan GmbH, Berlin-Buch, Germany). Then, 40 ml of this mixture was added to the Bioassay for GPCR-AAB measurement.

#### Influence of the aptamer BC 007 on the activities of GPCR-AABs

The aptamer BC 007 [10] is a single stranded 15 mer DNA oligonucleotide (5′-GGT TGG TGT GGT TGG-3′) (BioSpring GmbH, Frankfurt/Main, Germany).

To analyze the potency of BC 007 for the neutralization of GPCR-AABs present in dementia patients, the activity of the GPCR-AABs was measured in the bioassay performed in the absence and presence of 0.1 μM BC 007. To demonstrate the BC 007’s specificity for GPCR-AAB neutralization, experiments were performed in the presence of a scrambled 15 mer DNA aptamer (5′-GGT GGT GGT TGT GGT-3′) (BioTez Berlin-Buch GmbH, Germany).

#### Statistics

Statistical analysis was performed using the SPSS software package (SPSS Inc., Chicago, US) with Pearson chi-square test and Fisher's exact tests for the comparison of binary variables. For the intergroup comparison of continuous data, the Kruskal-Wallis H-test combined with the Mann-Whitney U-test for post-hoc analysis was employed. For this, undetectable marker concentrations (<lower limit of detection, LLD) were numerically expressed as values representing one-half of the LLD. For the graphical representation of continuous patient data, box plots indicate the median and interquartile range (IQR; 25th and 75th percentiles), while whiskers with ends represent the largest and smallest values inside 1.5 times the IQR, outliers (open circles) representing values between 1.5 and 3 times the IQR, and extremes (stars) placed more than 3 times the IQR.

## Results

### Patients’ basic data, group composition, comorbidities and medication

There were no significant differences in the subgroup composition related to the patients’ age and gender (Table 1). In all groups, significantly (p<0.001) more males vs. females were present. With respect to comorbidities and medication, significant differences between the dementia groups existed for the presence of hypertension and dementia drug medication. In detail, five patients with unclassified dementia had hypertension together with diabetes and coronary heart disease in two each. In Lewy body dementia, all patients presented with arterial hypertension, two had additionally coronary heart disease and two diabetes, one patient with coronary heart disease also had peripheral artery disease. In vascular dementia, all patients had hypertension, together with diabetes in 4 patients, coronary heart disease in 2 and peripheral artery disease in 2 patients. All patients with Alzheimer’s dementia presented with hypertension, 3 patients had additionally coronary heart disease and one patient peripheral artery disease. Patients with unclassified and vascular dementia did not receive any anti-dementia drugs, while it was prescribed for 5 patients with Lewy body dementia (Rivastigmine for 4 patients, Menmantine for 1 patient) and 7 patients with Alzheimer’s dementia (Donepezil in 3 patients, Memantine in 3 patients, and Galantamine in 1patient). Only two patients from the total cohort received immunotherapy at blood draw. Both patients had unclassified dementia and were taken oral prednisolone for suspected autoimmune contribution.

**Table 1:** Patients with unclassified, Lewy body, vascular and Alzheimer’s dementia. Group composition, patients’ basic data, comorbidities, and medication are demonstrated. (D.m., Diabetes mellitus; CHD, coronary heart disease; PAD, peripheral artery disease).

	Dementia					
Form		un-classified	Lewy body	vascular	Alzheimer’s	Significance
		(A)	(B)	(C)	(D)	(*) p <0.05
						(**) p<0.01
						(***) p<0.001
Group composition and basic data						
Number (n)		8	6	11	11	
Age (yrs)						
Median, Max/Min		63.5/77/45	70.5/78/67	72/81/55	70/87/40	
Male/Femal (n/n)		6/2	5/1	8/3	8/3	M vs. F (***)
(%/%)		75/25	83/17	73/27	73/27	
Comorbidities						
Hypertension (+/-)						Group diff. (*)
(n/n)		5/3	6/0	11/0	11/0	A vs.C p = 0.06
(%/%)		62.5/37.5	100/0	100/0	100/0	A vs. D p = 0.06
D.m. (+/-)						Group diff. (*)
(n/n)		5/3	2/4	4/7	0/11	A vs. D (**)
(%/%)		62.5/37.5	33/67	36/44	0/100	
CAD (+/-)						
(n/n)		2/6	2/4	2/9	3/8	
(%/%)		25/75	33/67	18/82	27/73	
PAD (+/-)						
(n/n)		0/8	1/5	2/9	1/10	
(%/%)		0/100	20/80	18/82	10/90	
Medication						
Antidementia drugs (+/-)						Group diff. (***)
(n/n)						A vs. B (**)
(%/%)		0/8	5/1	0/11	7/4	A vs. D (**)
		0/100	83/17	0/100	64/36	B vs. C (**)
Immuno-suppressiva (+/-)						
(n/n)						
(%/%)		2/6	0/6	0/11	0/11	
		25/75	0/100	0/100	0/100	

### The pattern of functional autoantibodies in patients with unclassified, Lewy body, vascular and Alzheimer’s dementia

The patients’ serum negativity and positivity, respectively, are demonstrated in Table 2 for α1-AABs, β2-AABs, and ETA-AABs. Negative patients were those with undetectable GPCR-AAB activities (<LLD) or with detectable activities but below the cut-off for separating healthy and diseased subjects; positive patients had GPCR-AAB activities >cut-off. Additionally, a score was calculated for the integrated activity assessment of the three GPCR-AABs. For the score calculation, the GPCR-AAB activities were summarized based on: 0 point = GPCR-AAB level <lower limit of detection (LLD); 1 point = GPCR-AAB level > LLD < cut-off; 2 points = GPCR-AAB level > cut-off.

**Table 2:** The pattern of functional autoantibodies in patients with unclassified, Lewy body, vascular and Alzheimer’s dementia. Serum positivity and negativity are demonstrated for autoantibodies directed against α1-adrenergic (α1-AABs), β2-adrenergic (β2-AABs) and endothelin A receptors (ETA-AABs); a score was calculated for the integrated activity assessment of the three autoantibodies directed against G-protein coupled receptors (GPCR-AABs). For the score calculation, the GPCR-AAB activities were summarized based on: 0 point = GPCR-AAB level <lower limit of detection (LLD); 1 point = GPCR-AAB level > LLD < cut-off; 2 points = GPCR-AAB level > cut-off.

	Dementia				
Form	un-classified	Lewy body	Vascular	Alzheimer’s	Significance
	(A)	(B)	(C)	(D)	(*) p <0.05
					(**) p<0.01
					(***) p<0.001
	Functional autoantibody pattern				
α1-AABs -/+					Group diff. (***)
(n/n)	6/2	5/1	2/9	1/10	A vs. C (*)
(%/%)	75/25	83/17	18/82	9/91	A vs. D (**)
					B vs. C (*)
					B vs. D (**)
β2-AABs -/+					Group diff. (p = 0.07)
(n/n)	2/6	5/1	3/8	3/8	
(%/%)	25/75	83/17	27/73	27/73	
ETA-AABs -/+					Group diff. (***)
(n/n)	7/1	5/1	1/10	9/2	A vs. C (*)
(%/%)	88/12	83/17	9/91	82/18	B vs. C (*)
					C vs. D (***)
1 AABs -/+					Group diff. (*)
(n/n)	2/6	4/2	1/10	1/10	B vs. C (*)
(%/%)	25/75	67/33	9/91	9/91	B vs. D (*)
2 AABs -/+					Group diff. (***)
(n/n)	6/2	5/1	1/10	2/9	A vs.C (**)
(%/%)	75/25	67/33	9/91	9/91	A vs.C (*)
					B vs. C (**)
					B vs. D (*)
3 AABs -/+					Group diff. (p = 0.07)
(n/n)	7/1	5/1	4/7	8/3	
(%/%)	88/12	83/17	36/64	73/27	
GPCR-AAB-Score (Median/Min/Max)					Group diff. (***)
	2/0/6	0/0/6	6/0/6	4/0/6	A vs. C (*)
					B vs. C (*)
					B vs. D (*)
					C vs. D (*)

Positivity for α1-, β2- and/or ETA-AABs was found in each form of dementia, but with significantly different frequencies. However, in the Lewy body dementia group, only one patient was positive for GPCR-AABs, who presented all three autoantibodies. In contrast, none of the study patients was positive for AT1-AABs.

Using the Pearson Chi-square test, we calculated frequency differences between the patient groups for α1-AABs (p<0.001) and ETA-AABs (p<0.001), whereas β2-AABs only tended (p = 0.07) to be different between the dementia forms. By post hoc analysis with Fisher’s exact test, we attributed the frequency differences for α1- and ETA-AABs specifically to the individual forms of dementia.

The majority of patients with vascular and Alzheimer’s dementia presented with α1- and β2-AABs; the vascular group also presented with ETA-AABs. Consequently, with respect to α1-AABs, significantly more patients were positive in the vascular (82%, p<0.05) and Alzheimer’s dementia (91%, p<0.01) groups than in the groups with unclassified (25%) and Lewy body dementia (17%). No significant differences existed between Alzheimer’s and vascular dementia or between unclassified and Lewy body dementia. ETA-AABs presented at a significantly higher frequency in patients with vascular dementia (91%) compared to those patients suffering from unclassified (12%, p<0.05), Lewy body (17%, p<0.05) or Alzheimer’s dementia (18%, p<0.001), who showed no differences.

Without reaching statistical significance, but numerically, the majority of patients with unclassified (75%), vascular (73%) and Alzheimer’s dementia (73%) presented with β2-AABs, whereas we found this autoantibody in only a minority of the patients with Lewy body dementia (17%).

To support any more pronounced autoimmune background in vascular and Alzheimer’s dementia compared with unclassified and Lewy body dementia, the patient cohort was analyzed for their general GPCR-AAB positivity as presented also in Table 1.

Significant group differences (Pearson Chi-square test) were calculated for the presence of at least one (p<0.05) or two (p<p<0.001) of the analyzed GPCR-AABs. In the case of the presence of three autoantibodies, a trend (p = 0.07) towards group differences was obvious. More detailed analyses (Fisher’s exact test) for the presence of at least any one or any two of the GPCR-AABs, showed significantly more patients with vascular (91%, p<0.05; 91%, p<0.01) and Alzheimer’s dementia (91%, 0.05; 91%, p<0.05) to be positive compared with the patients suffering from Lewy body dementia (33%). Related to the patients’ positivity for any one of the GPCRs, unclassified dementia (75%) did not differ from the other dementia forms. However, in cases with the presence of any two GPCR-AABs, significantly fewer patients with unclassified dementia (25%) were affected compared with the vascular (p<0.01) and Alzheimer’s dementia (p<0.05) patients. The more prominent role of GPCR-AABs in Alzheimer’s dementia and even more in vascular dementia was clearly supported by the score that based on the following assumption: GPCR-AABs level < LLD = 0 points; GPCR-AAB level > LLD < cut off = 1 point; GPCR-AAB level > cut off = 2 points. The vascular dementia form presented with a score that was significantly higher than that calculated for unclassified (p<0.05), Lewy body (0.05) and Alzheimer’s dementia (p<0.05). Additionally, the score for Alzheimer’s dementia was significantly higher than that of Lewy body dementia (p<0.05).

**Fig 3:**
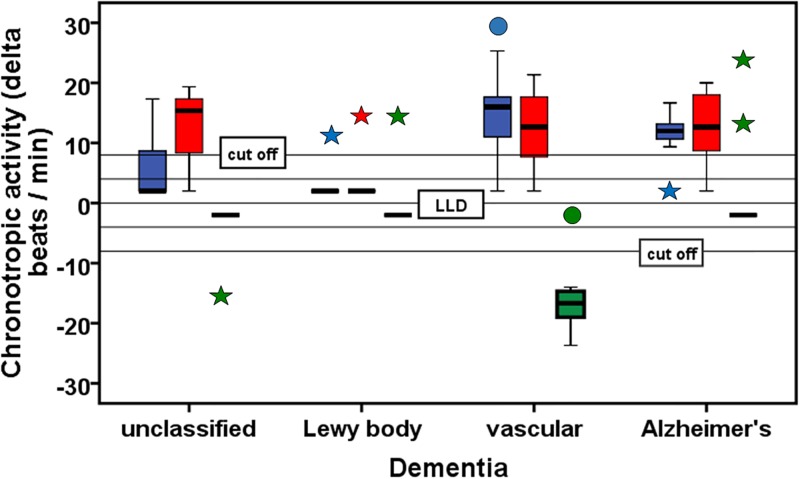
Activity of autoantibodies directed against the α1-adrenergic (blue), β2-adrenergic (red) and endothelin A receptor (green) in the serum of patients with unclassified, Lewy body, vascular and Alzheimer’s dementia. Box plots are plotted indicating median and interquartile range (IQR; 25th and 75th percentiles); whiskers have ends that represent the largest and smallest values inside 1.5 times the IQR, alongside outliers (open circles) that are values placed between 1.5 and 3 times the IQR, and extremes (stars) placed more than 3 times the IQR. Lower limit of detection (LLD) for positive and negative chronotropic activity = 4.0 U and -4.0 U; cut off for GPCR-AAB positivity = 8.0 U for positive and -8.0 U for negative chronotropic GPCR-AAB activity.

We have also proven whether the GPCR-AAB pattern in the dementia patients was determined by their comorbidities, but we did not see a significant relationship for any of the three GPCR-AABs. However using the Pearson chi-square and Fisher's exact test, we calculated for the presence of α1-AABs in hypertensive patients a p value of 0.051. Fig 3, presenting dementia group-related GPCR-AAB activities, substantiates the results of Table 1 and shows different α1- (p<0.005), β2- (p<0.05) and ETA-AAB activities (p<0.001) between the patients groups. Following post hoc analysis, the α1-AAB activity was higher in patients with vascular dementia than in those with unclassified (p<0.05) and Lewy body dementia (p<0.01) as well as in patients with Alzheimer’s dementia compared to Lewy body dementia (p<0.01). Lewy body dementia patients also presented with lower β2-AAB activity vs. patients with unclassified (p<0.05), vascular (p<0.05) and Alzheimer’s dementia (p<0.05). Significantly increased ETA-AAB activity was found exclusively in patients with vascular dementia vs. unclassified dementia (p<0.05), Lewy body dementia and Alzheimer’s dementia (p<0.01).

### Localization of the extracellular receptor binding side and mapping of the specific epitopes targeted by the GPCR-AABs

As indicated in Table 3, α1-AABs and ETA-AABs targeted the second extracellular loop of their receptors and the specific epitopes (^169^APEDET^174^) and (^234^EQHKTCMLNATSK^246^), respectively. β2-AABs were directed against the first extracellular loop specifically targeting the epitope (^101^FGNFWCE^107^). Compared with the epitope of α1-AABs and β2-AAB, the ETA-AAB epitope was clearly enlarged.

**Table 3:** Dementia-associated autoantibodies directed against G-protein coupled receptors (GPCR-AABs) such as those directed against the α1-adrenergic (α1-AABs), β2-adrenergic (β2-AABs) and endothelin A receptor (ETA-AABs) related to their target (extracellular receptor loop) with the specific epitope.

GPCR-AABs	Extracellular receptor loop	Specific epitope
α1-AABs	Loop II	APEDET
β2-AABs	Loop I	FGNFWCE
ETA-AABs	Loop II	EQHKTCMLNATSK

**Fig 4:**
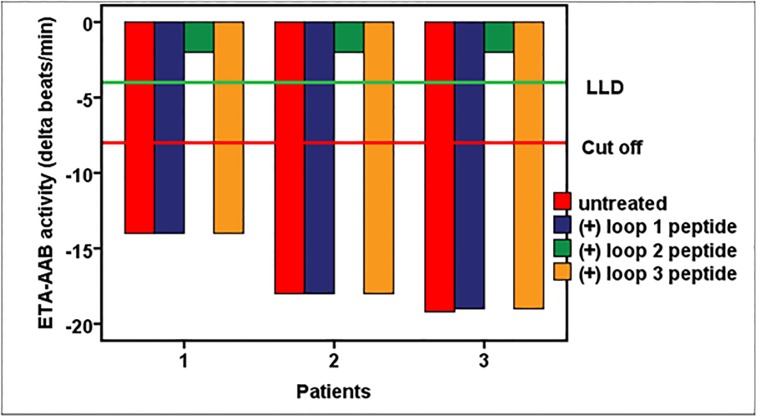
Autoantibodies directed against the endothelin A receptor (ETA-AABs) of patients with vascular dementia target the second extracellular receptor loop. Using the bioassay of spontaneously beating cultured neonatal rat cardiomyocytes, the chronotropic activity of the patients’ IgG (n = 3), either untreated or pre-incubated with peptides representing the first (^134^LPINVFKLLAGRWPFDHNDFGVFLCKL^160^), second (^229^FEYRGEQHKTCMLNATSKFMEFYQDVKD^256^), and third extracellular receptor loop (^329^KKTVYNEMDKNRCELLLSFLL^348^), was measured. Values below the low limit of detection (LLD) were displayed as half range values. LLD = -4 beats/min; cut-off (separating healthy from disease subjects) = − 8 beats per/min.

**Fig 5:**
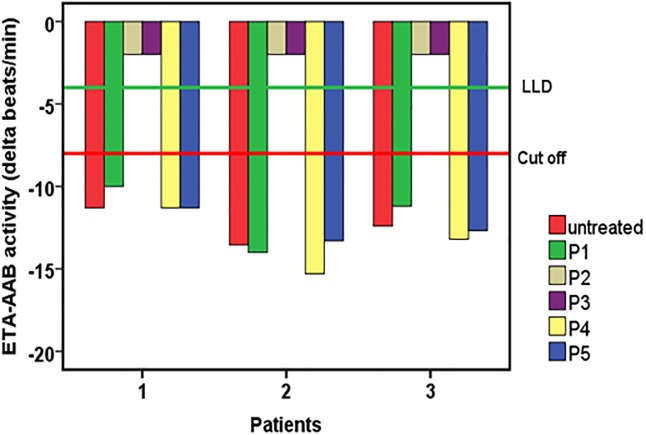
Mapping of the second extracellular loop of the endothelin A receptor for epitope localization targeted by autoantibodies directed against the endothelin A receptor (ETA-AABs) of patients with vascular dementia. Using the bioassay of spontaneously beating cultured neonatal rat cardiomyocytes, the ETA-AAB activity of the patients’ IgG (n = 3) untreated or pre-incubated with peptides that overlapped to represent the second extracellular endothelin A receptor loop (P1: FEYRGEQ, P2: EQHKTCM, P3: MLNATSK, P4: SKFMEFY, P5: FYQDVKD) was measured. Values below the low limit of detection (LLD) were displayed as half range values. LLD = − 4 beats/min; cut-off (separating healthy from disease subjects) = − 8 beats per/min.

For the first described ETA-AABs, the *in vitro* experiments to localize their extracellular binding sites and the specific epitope are demonstrated in Figs 4 and 5.

### Influence of the aptamer BC 007 on the activity of a1-AABs, β2-AABs and ETA-AABs of patients with dementia

**Fig 6:**
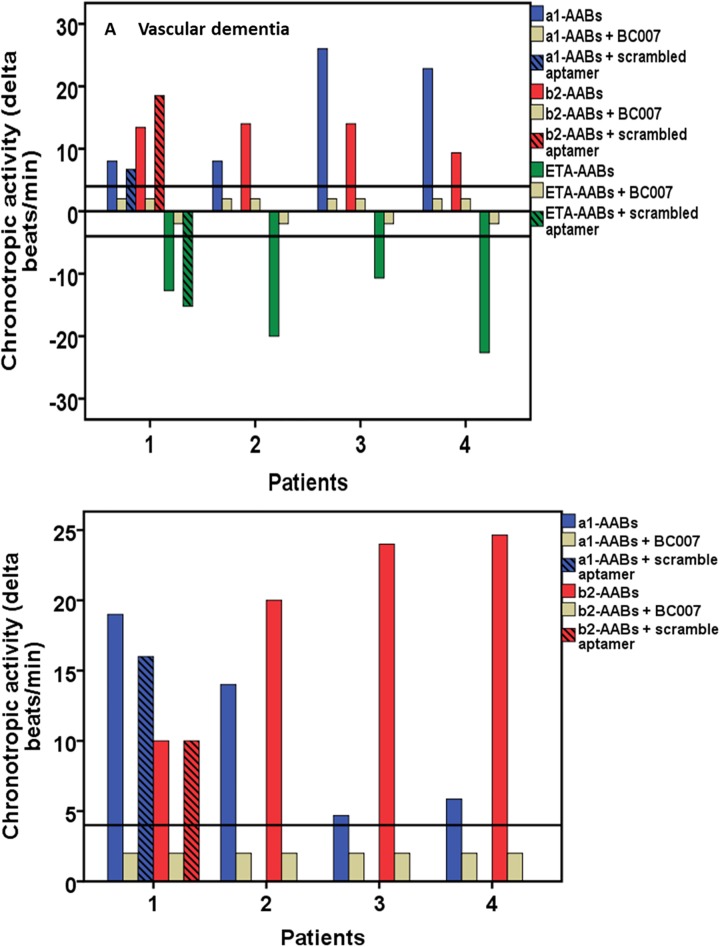
A and B. Influence of the aptamer BC 007 on the activity of autoantibodies directed against the G-protein coupled receptors (GPCR-AABs) specifically those to the β2-adrenergic (β2-AABs), α1-adrenergic (a1-AABs) and endothelin A receptor (ETA-AABs) in patients with (A) vascular (β2-AABs, α1-AABs, ETA-AABs) and (B) Alzheimer’s dementia (β2-AABs, α1-AABs). The total chronotropic activity of the patients’ IgG as well as the activities related to each autoantibody on spontaneously beating cultured neonatal rat cardiomyocytes isolated from the serum of all 4 patients in the absence (colored columns) and presence (0.1 μM) of BC 007 (grey columns) are demonstrated. For each one of the patients with vascular and Alzheimer’s dementia, the GPCR-AAB activity in the presence of a scrambled 15 mer aptamer is additionally demonstrated. Values below the low limit of detection (black line = LLD) were displayed as half range values. LLD = 4 beats/min and—4 beats/min, respective.

Fig 6A and 6B show representative results for 4 patients positive for α1-, β2-and additionally ETA-AABs who suffered from vascular dementia and for 4 patients positive for α1- and β2-AABs who suffered from Alzheimer’ dementia. Their GPCR-AAB activities in the absence and presence (0.1 μmol/l) of the aptamer BC 007 are demonstrated. For two patient, one with vascular dementia and another with Alzheimer’s dementia, the GPCR-AAB activity was measured in the presence of the scrambled 15 mer aptamer. In the presence of BC 007, no GPCR-AAB activity could be measured. In contrast, the scrambled aptamer did not inhibit the activity of any of the dementia associated GPCR-AABs.

## Discussion

A distinct proportion of patients with dementia are immunotherapy-responsive [16], which indicates a dementia-related autoimmune background. Target-destructing autoantibodies, many of which are documented in [1,2], were preferentially discussed in this context. However, there is a new class of functional autoantibodies which bind to GPCRs. Uncontrolled long-lasting receptor over-stimulation, which induces pathologically relevant disturbances in cell morphology and function, are the consequence as summarized in [3,4,5].

Our study demonstrates for the first time that serum antibodies against the ETA-R are relatively common in patients with vascular dementia. ETA-AABs have already been found in patients with pulmonary hypertension [17], scleroderma [18], thromboangiitis obliterans [19] and benign prostate hyperplasia [20]. The ETA-AABs of patients with dementia targeted a more terminal epitope located on the second extracellular receptor loop, which is different from the epitope targeted by the ETA-AABs in benign prostate hyperplasia [20]. For the ETA-AABs’ pathophysiologic function, their pathology driving or at least supporting role was intensively discussed for systemic scleroderma [18]. For brain pathology, the negative influence of endothelin was documented on neuron and blood-brain barrier integrity [21], synaptic plasticity [22], oxidative stress and apoptosis [23]. Following ETA-R blockade, protective effects such as lower degree of impaired learning and memory have been evidenced in animals [24,25]. The authors argued that ETA-R blockade diminishes the vasoactive effects of endothelin and this way in which ischemia damages the hippocampus, which is responsible for learning and memory. Considering the high interchangeability in the pathogenic effects of endothelin A and ETA-AABs, we assume that ETA-AABs in patients with vascular dementia could contribute to the pathogenesis whereby the ETA-AABs pathogenicity could exceed that of endothelin due to the absence of control mechanisms (receptor desensitization, receptor down regulation) to counteract over-boarding receptor stimulation. Only a minority of patients in the other groups presented with ETA-AABs. It remains speculative whether this indicates any non-recognized vascular background in these patients. Patients positive for ETA-AABs, independent of their clinically diagnosed dementia form, were always also positive for α1- and β2-AABs (except for one patient with Alzheimer’s dementia). Consequently, these patients presented with the typical vascular dementia GPCR-AAB pattern.

The presence of α1- and β1-AABs in patients with vascular and Alzheimer’s dementia confirmed previous studies [7,8]. Just as for the ETA-AABs, uncontrolled and over-boarding receptor stimulation were discussed as the key event for the autoantibodies’ pathogenic potency. However, as summarized in [26], the brain noradrenergic system plays a pivotal role in modulating cognitive activities. Consequently, increased agonist availability resulted in improved cognitive activities, whereas deficits in cases of low agonist levels have been documented. It is tempting to speculate that autoantibody-dependent over- and uncontrolled stimulation of the adrenergic system could disturb this highly regulated neurotransmission via the physiologic agonists leading to cognitive dysfunction.

Furthermore, β2-adrenergic receptor stimulation increased gamma-secretase activity for accelerated amyloid plaque formation, being one of the hallmarks in Alzheimer’s disease [27]

To attribute the place of α1- and β2-AABs in the orchestra of pathogenic players in vascular dementia, there are some points of discussion. The α1-AABs’ potency for inducing cellular remodeling processes has been demonstrated in rat heart [28] and vessels [29], (cardiomyocyte hypertrophy, aortic media thickening, collagen deposition in heart interstitium, mitochondria hyperplasia of vascular smooth muscle cells, increased expression of c-jun and matrix metalloproteinases). Many of these events were also accused of promoting vascular alterations in dementia patients [30,31]. Vascular defects in the brain have been evidenced by magnetic resonance imaging in rats immunized for the generation of α1-AAB [32]. Related to β2-AR stimulation, vascular pathology with enhanced vasoconstrictor response and increased vascular oxidative stress has been demonstrated, which might result in endothelial dysfunction [33].

The α1-AABs targeted the second, the β2-AABs the first extracellular receptor loop of the related receptors. This—together with the identified epitopes—confirmed the data in [7] but was—concerning the receptor loop for α1-AABs—in contrast to the data in [8] where α1-AABs targeted the first loop. We cannot absolutely exclude first loop targeting α1-AABs in dementia patients, however, we haven’t seen such. In contrast to a recently published study, which used ELISA to demonstrate AT1-AAB positivity in Alzheimer’s patients [9]; the bioassay didn’t display functional active AT1-AABs in our patients. However, ELISA cannot distinguish between functional active and inactive GPCR-AABs and is therefore under criticism if not supplemented and validated with a functional assay such as the here used bioassay [4, 34, 35]. Although this criticism focused preferentially to ELISA vs. bioassay measurement of autoantibodies directed against the beta1-adrenergic receptor, the same problem exists in our view for all the other GPCR-AABs.

To connect serum GPCR-AABs with pathological processes in the brain, the AABs’ or related B-cells’ crossing of the blood-brain barrier would be prerequisite. As summarized in [36,37,38,39], there is no doubt that routes exist for the pathogenic autoantibodies and related B-cells to attack the “immune-privileged” central nervous system. Consequently, for dementia patients, treatment strategies (therapeutic plasma exchange (TPE), immunoadsorption) for GPCR-AAB removal from the patient’s circulation could be promising and have already tested as extensively reviewed for GPCR-AAB positive heart failure patients [5,6]. Recently, immunoadsorption was also tested in eight α1-AABs positive patients (5 patients were additionally positive for β1-AABs) with vascular/Alzheimer’s dementia [40]. Patients who completed the aforementioned cycle immunoadsorption protocol, demonstrated nearly 100% α1-AAB removal; no autoantibody returns within the follow up period of 18 months (except one patient with GPCR-AAB return after 12 month) combined with the stabilizing of the Mini-Mental State Examination Score (MMES) at the level before treatment. In contrast, MMES deteriorated in the patients who interrupted the immunoadsorption protocol after the second and third run, respectively, therefore having incomplete α1-AAB removal and autoantibody return over time. Based on their findings, the authors hypothesized a substantial role of α1-AABs in the pathogenesis of dementia, specifically of Alzheimer’s and vascular dementia.

We are rather reserved to agree any exclusive or dominant pathogenic role specifically of α1-AABs in dementia. α1-AABs are typically associated to hypertension [41,42] and could therefore—as demonstrated for our dementia patient cohort—related to this frequent dementia comorbidity. However, the immunoadsorption technology used procided a design for the removal of the whole IgGs. Consequently, the patients’ blood was cleared from α1-AABs and all of the other possibly pathogenic IgG-associated AABs; in case of the dementia patients, therefore, also from β2-AABs and ETA-AABs. Perhaps because of this, unspecific immunoadsorption for removal of all the GPCR-AABs in dementia patients should be a more hopeful treatment option than treatment concepts directed specifically to a one of the GPCR-AABs. Unfortunately, cost factors, logistical problems and patient’s burden are associated with immunoadsorption, which form the main reasons for its restricted use. Treatment strategies for *in vivo* GPCR-AAB attack would minimize these problems and therefore be superior. Although already studied in dementia patients [43], intravenous IgG treatment (IVIG) and B-cell depletion were until now not applied specifically to attack the GPCR-AABs.

In the case of further manifestation of the GPCR-AABs pathogenic role in dementia patients, the *in vitro* neutralization of all three dementia-associated GPCR-AABs by the aptamer BC007 offer, as here demonstrated principally, a new treatment strategy. BC 007 is a single stranded 15 mer DNA oligonucleotide (5′-GGT TGG TGT GGT TGG-3′) that was patented for the use as GPCR-AAB “broad spectrum neutralizer in diseases associated with GPCR-AABs. As we recently demonstrated [44], BC007 binds to the GPCR-AABs Fab fragments but clearly outside the complementarity-determining regions (CDRs) which explains the “surprising” potency of BC 007 for the neutralization of most several GPCR-AABs. Therefore most important, BC007 is able to also neutralize pathogenic GPCR-AABs directed to either other receptor loops or even other G-protein coupled receptors. This could be helpful for dementia patients suffering from comorbidities positive for other GP [10,44,45] CR-AABs (e.g. hypertension, diabetes mellitus) and interfering and aggravating dementia. In our view, this makes BC007 superior for treatment compared to compounds, which bind and affect the GPCR-AABs CDRs. To achieve such a concept, for each GPCR-AAB present in patients, a specific drug would be necessary. Irrespective of this disadvantage of CDR-binding compounds, a cyclic peptide [46] and an aptamer [47] were suggested for neutralization of the second loop targeting β1-AABs for the treatment of patients with DCM. However due to DCM patients with pathogenic β1-AABs directed against the first receptor loop and the frequent co-presentation of DCM patients with β1-AABs and GPCR-AABs directed against the muscarinic 2 receptor, α1-adrenergic and angiotensin 1 receptor type 2 in DCM patients, BC007 should be also superior in the treatment of these patients.

Translation of the aptamer-dependent concept of *in vivo* GPCR-AAB neutralization into clinical trials with GPCR-AAB-positive dementia patients might be a longer way. However related to autoimmunity-compromised heart failure patients, specifically those with DCM, promising steps such as animal studies [45], pre-clinical investigations as well as the phase I clinical trial [48] were already taken.

## Conclusion

Patients with vascular and Alzheimer’s dementia, and to a lower frequency unclassified dementia, were carriers of α1- and β2-AABs. Patients suffering from vascular dementia carry additional ETA-AABs, which agrees with the strong vascular pathology in these patients. While α1- and ETA-AABs targeted specific epitopes on the second extracellular receptor loops, β1-AABs were directed against the first receptor loop. The majority of patients with Lewy body dementia were free of GPCR-AABs.

Due to the finding of GPCR-AABs in dementia patients, specifically in those suffering from Alzheimer’s and vascular dementia, the orchestra of players responsible for the autoimmune background in these dementia forms, which was so far preferentially represented by the classic autoantibodies, should be supplemented by functional autoantibodies. Because the functional autoantibodies found in dementia patients could be neutralized *in vitro* by the aptamer BC007, the first step was taken towards a new *in vivo* treatment option in GPCR-AAB-positive dementia patients.

## Supporting information

S1 Table Row data of Tables 1 and 2 and Fig 3. (XLSX)

S2 Table Row data of Fig 2. (XLSX)

S3 Table Row data of Figs 4 and 5. (XLSX)

S4 Table Row data of Fig 6A. (XLSX)

S5 Table Row data of Fig 6B. (XLSX)
