# Correction: Functional autoantibodies in patients with different forms of dementia

**DOI:** 10.1371/journal.pone.0203253

**Published:** 2018-08-27

**Authors:** Gerd Wallukat, Harald Prüss, Johannes Müller, Ingolf Schimke

There is an error in the sixth sentence of the Abstract. The correct sentence is: Patients with Alzheimer’s and vascular dementia carried GPCR-AABs targeting the second loop of the alpha1- and the first loop of the beta2-adrenergic receptors (α1-AABs; β2-AABs).

There is an error in the third sentence of the Conclusion. The correct sentence is: While α1- and ETA-AAB targeted specific epitopes on the second extracellular receptor loops, β1-AABs were directed against the first receptor loop.
